# Incidence of anxiety and depression in children and young people with life-limiting conditions

**DOI:** 10.1038/s41390-022-02370-8

**Published:** 2022-11-11

**Authors:** Mary M. Barker, Bryony Beresford, Lorna K. Fraser

**Affiliations:** 1 Diabetes Research Centre, University of Leicester, Leicester General Hospital, Gwendolen Road, Leicester, UK; 2 Martin House Research Centre, Department of Health Sciences, University of York, York, UK; 3 Social Policy Research Unit, University of York, York, UK

## Abstract

**Background:**

The aim of this study was to investigate the incidence of anxiety and depression in children and young people with life-limiting conditions.

**Methods:**

A comparative cohort study was conducted, using primary and secondary care data from the Clinical Practice Research Datalink (CPRD) in England. Anxiety and depression codes were identified using diagnostic, symptom and prescription codes. Incidence rates of anxiety and depression were compared across condition groups using Poisson regression, adjusting for sex, age, ethnicity, and deprivation status.

**Results:**

A total of 25,313 children and young people were included in the study: 5527 with life-limiting conditions, 6729 with chronic conditions, and 13,057 with no long-term conditions. The incidence of anxiety (IRR_adj_: 1.39, 95% CI: 1.09–1.77) and depression (IRR_adj_: 1.41, 95% CI: 1.08–1.83) was significantly higher in children and young people with life-limiting conditions, compared to children and young people with no long-term conditions.

**Conclusions:**

The higher incidence of anxiety and depression observed among children and young people with life-limiting conditions highlights the need for psychological support in this population, including further efforts to prevent, identify, and treat anxiety and depression.

**Impact:**

- The analysis of primary and secondary healthcare data from England revealed that the incidence of anxiety and depression was higher among children and young people with life-limiting conditions, compared to those with no long-term conditions.
- This is the first study to investigate the incidence of anxiety and depression in children and young people with a wide range of life-limiting conditions.
- The higher incidence of anxiety and depression observed in children and young people with life-limiting conditions highlights the need for psychological support aiming to prevent, identify, and treat anxiety and depression in this population group.

## Introduction

Mental health conditions, the leading cause of disability, affect 10–20% of children and adolescents globally.^1,2^ These conditions often persist into adulthood, with 75% of long-term mental health conditions observed among adults emerging before the age of 24 years.^3^ Anxiety and depression are particularly common, experienced by 6.5 and 2.6% of children and young people, respectively, worldwide.^4^ The presence of a chronic physical condition has been shown to increase the risk of anxiety and depression in children and young people.^5–8^ Life-limiting conditions, a type of chronic physical condition, are those which are likely to cause death in children and young people (e.g. Batten disease) or for which curative treatments are available but may result in failure (e.g. cancer).^9^ Children and young people living with any chronic condition face various challenges, such as painful and distressing symptoms and side-effects from treatment, in addition to complex disease management regimens.^10,11^ Frequent medical appointments and hospitalisations can result in missed schooling, potentially disrupting both their education and relationships with their peers.^10^ All of these challenges can also contribute to children and young people feeling different from their peers, leading to feelings of embarrassment and social isolation.^12,13^ Illness uncertainty is another major challenge.^12,14^ However, a crucial and additional challenge experienced by children and young people living with a life-limiting condition is the potential for fears relating to the possibility or certainty of premature death.^15,16^ The presence of these additional challenges suggests that they could be even more at risk from anxiety and depression than children and young people with non-life-limiting chronic conditions. This, in addition to the rising prevalence of life-limiting conditions in England in the past two decades,^17^ necessitates research investigating the mental health of children and young people in this population.

A recent systematic review and meta-analysis of 37 studies, found a markedly higher prevalence of anxiety and depression among children and young people with life-limiting conditions compared to findings from the general population of children and young people.^18^ However, the existing studies reviewed in the meta-analysis included children and young people with a very narrow range of specific life-limiting conditions, whilst also under-representing children and young people with neurological conditions. In addition, no research into the incidence of anxiety and depression in children and young people with life-limiting conditions has previously been conducted. This study aimed to investigate the incidence of anxiety and depression in children and young people with life-limiting conditions, compared to children and young people with chronic conditions which are not life-limiting, and those without a long-term condition. The factors associated with anxiety/depression incidence in each condition group were also explored.

## Methods

This study is reported according to Strengthening the Reporting of Observational Studies in Epidemiology (STROBE)-RECORD guidelines.^19^

### Data sources

Data for this study were sourced from the Clinical Practice Research Datalink (CPRD) GOLD dataset, a longitudinal primary care dataset. In the UK, doctors (known as general practitioners (GPs)), along with a team of other trained professionals, are the providers of primary care, and CPRD contains records from a nationally representative sample of over 1900 GP practices from across the UK.^20,21^ CPRD data is deterministically linked to secondary care data from Hospital Episodes Statistics (HES) in England and death registration data from the Office for National Statistics (ONS). The CPRD GOLD dataset contains data regarding primary care consultations, diagnoses, referrals and prescriptions, in addition to sociodemographic data such as age, sex, and ethnicity. HES data include information relating to diagnoses and clinical procedures, as well as patient demographics.

Approval for the use of CPRD data in this study was granted from CPRD’s Independent Scientific Advisory Committee (ISAC) (protocol ref.: 16_877).

### Study population

The cohort used in this study was identified by CPRD (Supplementary Fig. 1). The classification of life-limiting and chronic conditions was conducted using previously developed Read code (CPRD) and ICD-10 code (HES) lists.^22,23^ Children and young people with life-limiting conditions, the index group in the cohort identification process, were included if they were registered at a CPRD practice between 1 April 2007 and 31 December 2017 and were eligible for linkage. These children and young people were subsequently matched on year if birth, sex, and region to one child or young person with a chronic condition, and up to two children with no long-term condition. Children were eligible for the study if they were aged between 5 and 18 years.

The index date for the study was set as the latest of the child or young person’s diagnosis of a life-limiting condition/chronic condition and the date at which they turned 5 years old. Participants were followed up until the earliest of the following: occurrence of the outcome (anxiety/depression), date at which they turned 19 years old, date of death, date they transferred out of the practice, last data collection date of the practice and the study end date (31 December 2017). Children and young people with a record of the outcome of interest before the date at which they were diagnosed with a life-limiting/chronic condition were excluded from the analysis for that outcome. In addition, the first month of registration at the practice was excluded from the follow-up time, as codes recorded during this period are often related to prevalent, rather than incident, conditions.^24^

### Outcomes

Anxiety and depression were identified using diagnostic and symptom Read codes in CPRD, prescription codes in CPRD, and ICD-10 codes in HES. Lists of diagnostic and symptom Read codes were generated using previously published methods.^25^ All ICD-10 codes relating to anxiety or depression, except for one code for specific (isolated) phobias and one code relating to co-morbid anxiety and depression, were included in each respective code list. Lists of suicide-related Read and ICD-10 codes were used to aid in the identification of depression cases. Read codes relating to the use of anxiety/depression screening tools were also included, in addition to generic codes from CPRD or HES data which indicated the offer or receipt of generic psychological support. Previously published methods were also used to generate lists of anxiety/depression prescription codes; namely, prescriptions for antidepressants, anxiolytics, and hypnotics.^25^

**Fig. 1:**
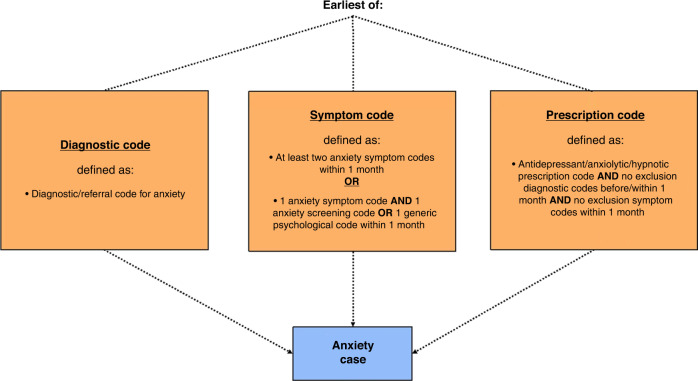
Anxiety case definition algorithm. Algorithm showing the codes/combinations of codes and associated time restrictions used to define a case of anxiety.

**Fig. 2:**
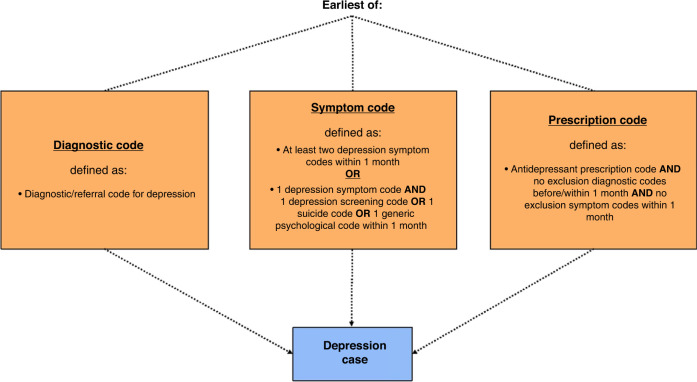
Depression case definition algorithm. Algorithm showing the codes/combinations of codes and associated time restrictions used to define a case of depression.

Algorithms were developed to define the criteria of codes which needed to be recorded in order for a case of anxiety/depression to be assigned (Figs. 1 and 2). This was necessary due to the varying level of ambiguity related to each type of code. Diagnostic codes for anxiety/depression are highly specific, meaning that the recording of one of these codes very likely represents a case of anxiety/depression. This is also similar for referral codes (all termed ‘diagnostic codes’ hereafter for simplicity).

Symptom codes, however, are more ambiguous, including symptoms such as ‘nerves’ and ‘fear’ for anxiety. A previous study investigating the incidence of anxiety/depression only included symptoms codes in the analysis if they were coupled with a prescription code for anxiety/depression, in order to increase the specificity of the analysis.^26^ However, as the use of antidepressants in children and young people is cautioned, the current study employed a different method previously used in a study of depression incidence among adolescents.^24^ Hereby, a case of anxiety/depression was assigned if two anxiety/depressive symptoms were recorded within 1 month. As the results of anxiety/depression screening tools were not included in the data, a case of anxiety/depression was only assigned if the screening tool code was coupled with a symptom code within 1 month. The same principle was used for generic psychological codes and suicide-related codes. All of the above codes will hereafter be referred to as ‘symptom codes’ for simplicity.

Similarly, prescription codes also needed to be studied in accordance with the other codes recorded in the child or young person’s record, due to the various indications for which the drugs can be prescribed. Importantly, many of the indications for which anxiolytics, hypnotics, and some antidepressants are prescribed occur far more frequently among children and young people with life-limiting conditions. For example, Diazepam is prescribed for indications such as muscle spasms, in addition to anxiety. Therefore, the inclusion of all anxiolytics, hypnotics, and antidepressants could have resulted in biased estimates of anxiety/depression incidence which were not comparable across condition groups. Consequently, drugs were excluded from this study if one or more of the following applied: the drug was predominantly prescribed for children and young people with life-limiting conditions, very few children and young people prescribed the drug also had a recorded diagnosis or symptom code for anxiety/depression, or the drug has multiple indications other than anxiety/depression. The analysis relating to these drug eligibility criteria is displayed in Supplementary Tables 1–3. Finally, to further improve the specificity of the analysis, a list of exclusion codes relating to the main indications of each drug (other than anxiety/depression) was compiled. Using this list, prescription codes were only used to assign a case of anxiety/depression if no exclusion diagnostic codes were recorded before or within one month of the prescription code, and no exclusion symptom codes were recorded within one month of the prescription code.

### Confounders

Sex, age, ethnicity, and deprivation status were included as confounders in the analysis. Participants’ age was calculated as their age at entry to the study. Ethnicity data was available from the HES dataset, and was set for each participant as their most frequently recorded ethnicity category, excluding missing. Six ethnic categories were used for the main analysis of the study: White, Black, South Asian, Other Asian, Mixed and Other. Due to small group numbers in the stratified analysis, all ethnicities other than White were merged to create one ‘Other’ ethnic category. Deprivation status was assessed using linked Index of Multiple Deprivation 2010 (IMD 2010)^27^ data for the most recent known address of each participant. Deprivation status was categorised into five groups, ranked from category 1 (least deprived) to category 5 (most deprived).

### Statistical analysis

Crude incidence rates of anxiety and depression were calculated for the overall study sample, and for each condition group, by dividing the number of incident cases by the total person-time at risk. Poisson regression modelling was used to generate incidence rate ratios (IRRs) comparing the incidence of each outcome between condition groups, accounting for time at risk. Both univariate and multivariable models (adjusting for sex, age, ethnicity, and deprivation status) were used. Stratified analysis, by condition group, was also conducted in order to explore the associations between potential risk factors (sex, age, ethnicity, and deprivation status) and anxiety/depression incidence. All analyses were conducted using STATA v.16.1.

## Results

The total study sample included 25,313 children and young people; 5527 with life-limiting conditions, 6729 with chronic conditions, and 13,057 with no long-term conditions. The sociodemographic characteristics of the included participants are displayed in Table 1. Over half (56.2%) of the participants were male, and the median age at study start was 5.00 years (IQR: 5.00–9.05 years). Low levels of missing data were found for all variables except ethnicity, for which 9.5% of children and young people had missing data.

**Table 1:** Participant characteristics.

Variable	Condition group			Total (*n* = 25,313)
	Life-limiting condition (*n* = 5527)	Chronic condition (*n* = 6729)	No long-term condition (*n* = 13,057)	
Age at study entry (years), median (IQR)	5.33 (5.00–9.94)	5.67 (5.00–9.79)	5.00 (5.00–8.38)	5.00 (5.00–9.05)
Sex, *n* (%)				
Male	3128 (56.6)	3778 (56.2)	7311 (56.0)	14,217 (56.2)
Female	2399 (43.4)	2951 (43.8)	5746 (44.0)	11,096 (43.8)
Ethnicity, *n* (%)				
White	4720 (85.4)	5519 (82.0)	9993 (76.5)	20,232 (79.9)
South Asian	256 (4.6)	280 (4.2)	354 (2.7)	890 (3.5)
Black	133 (2.4)	170 (2.5)	286 (2.2)	589 (2.3)
Other Asian	77 (1.4)	77 (1.1)	143 (1.1)	297 (1.2)
Mixed	130 (2.4)	164 (2.4)	280 (2.1)	574 (2.3)
Other	72 (1.3)	86 (1.3)	168 (1.3)	326 (1.3)
Unknown/missing	139 (2.5)	433 (6.4)	1833 (14.0)	2405 (9.5)
Deprivation status, *n* (%)				
1 (least deprived)	1218 (22.0)	1602 (23.8)	3582 (27.4)	6402 (25.3)
2	1177 (21.3)	1354 (20.1)	2709 (20.8)	5240 (20.7)
3	1058 (19.1)	1294 (19.2)	2411 (18.5)	4763 (18.8)
4	1077 (19.5)	1305 (19.4)	2293 (17.6)	4675 (18.5)
5 (most deprived)	995 (18.0)	1171 (17.4)	2052 (15.7)	4218 (16.7)
Missing	≤10	≤10	≤10	15 (0.1)

Cell values of 10 or less are censored (≤10).

### Incidence of anxiety

**Fig. 3:**
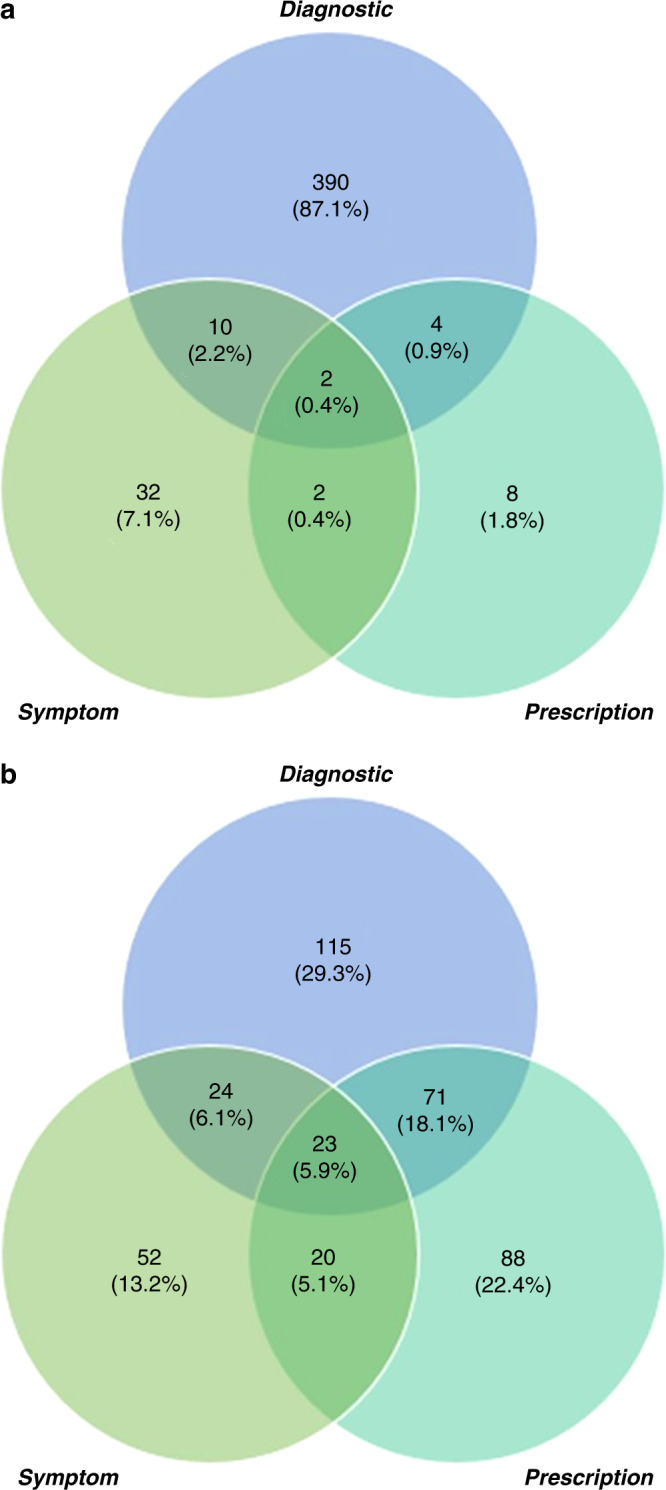
Code types recorded for children and young people with incidence anxiety and depression. Venn diagrams showing the types of codes recorded for children and young people with incident (**a**) anxiety and (**b**) depression.

Of the 25,313 children and young people in the study sample, 132 had been diagnosed with anxiety prior to the start of the study and were excluded from the analysis of anxiety incidence. Therefore, 25,181 children and young people were included, providing a total follow-up time of 122,157.66 person-years, with each child or young person contributing a median of 4.57 (IQR: 2.21–7.23) years of follow-up time. In total, 448 (1.78%) of children and young people had an incident case of anxiety (Table 2). The types of anxiety codes reported are displayed in Fig. 3a, showing that the majority (90.6%) of children and young people with incident anxiety had a diagnostic anxiety code recorded.

**Table 2:** Incidence of anxiety, by condition group.

	Number of children	Anxiety cases, no. (%)	Person-years in 1000s	Anxiety incidence rate per 1000 person-years (95% CI)	Crude incidence rate ratio (95% CI)	Adjusted incidence rate ratio (95% CI)
Total sample	25,181	448 (1.78)	122.16	3.67 (3.34–4.02)	NA	NA
Condition group						
No long-term condition	13,022	195 (1.50)	68.36	2.85 (2.48–3.28)	1.00 (ref)	1.00 (ref)
Chronic condition	6679	152 (2.28)	30.17	5.04 (4.30–5.91)	1.77 (1.43–2.18)**	1.64 (1.32–2.03)**
Life-limiting condition	5480	101 (1.84)	23.63	4.27 (3.52–5.20)	1.50 (1.18–1.91)**	1.39 (1.09–1.77)**

Adjusted incidence rate ratio adjusted for age, sex, ethnicity, and deprivation status.

*NA* not applicable.

**p ≤ 0.01.

**Fig. 4:**
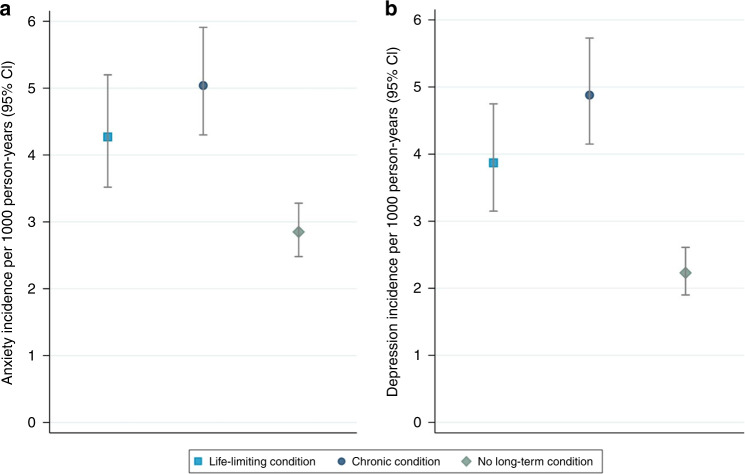
Incidence rates of anxiety and depression, stratified by condition group. Incidence rates of (**a**) anxiety and (**b**) depression in children and young people with life-limiting conditions (blue square), chronic conditions (blue circle) and no long-term conditions (green diamond).

The crude incidence rate of anxiety was highest in the chronic condition group (5.04 cases per 1000 person-years, 95% CI: 4.30–5.91), followed by the life-limiting condition group (4.27 cases per 1000 person-years, 95% CI: 3.52–5.20). The lowest anxiety incidence was observed in the no long-term condition group (2.85 cases per 1000 person years, 95% CI: 2.48–3.28) (Fig. 4a). In both the unadjusted and adjusted models, the anxiety incidence rate ratio was significantly higher in the life-limiting condition group (IRR_adj_: 1.39, 95% CI: 1.09–1.77) and the chronic condition group (IRR_adj_: 1.64, 95% CI: 1.32–2.03) compared to the no long-term condition group (Table 2). The full results from the adjusted model can be found in Supplementary Table 4.

Increased age and female sex were significantly associated with anxiety incidence in all three condition groups. Although a higher incidence of anxiety was observed among children and young people from the Other ethnic group, compared to the White ethnic group, in all condition groups, this was not statistically significant. No association was found between deprivation status and anxiety incidence in any of the condition groups (Table 3).

**Table 3:** Stratified analysis of potential risk factors for anxiety incidence, by condition group.

	Incidence rate (per 1000 person-years) (95% CI)	Crude incidence rate ratio (95% CI)	Adjusted incidence rate ratio (95% CI)
*Life-limiting condition group*			
Age	NA	1.14 (1.08–1.19)**	1.14 (1.08–1.20)**
Sex			
Male	3.12 (2.31–4.21)	1.00 (ref)	1.00 (ref)
Female	5.89 (4.55–7.62)	1.89 (1.27–2.80)**	1.73 (1.15–2.59)**
Ethnicity			
White	4.36 (3.54–5.36)	1.00 (ref)	1.00 (ref)
Other	2.35 (1.06–5.23)	0.54 (0.24–1.23)	0.67 (0.29–1.54)
Deprivation status			
1 (least deprived)	5.31 (3.69–7.64)	1.00 (ref)	1.00 (ref)
2	4.22 (2.78–6.40)	0.79 (0.46–1.38)	0.84 (0.48–1.49)
3	3.77 (2.34–6.06)	0.71 (0.39–1.29)	0.74 (0.40–1.37)
4	3.40 (2.05–5.63)	0.64 (0.34–1.19)	0.64 (0.33–1.23)
5 (most deprived)	4.48 (2.83–7.12)	0.84 (0.47–1.52)	0.82 (0.44–1.54)
*Chronic condition group*			
Age	NA	1.14 (1.09–1.18)**	1.13 (1.08–1.18)**
Sex			
Male	3.58 (2.79–4.58)	1.00 (ref)	1.00 (ref)
Female	7.09 (5.76–8.73)	1.98 (1.44–2.74)**	1.92 (1.38–2.67)**
Ethnicity			
White	5.53 (4.68–6.54)	1.00 (ref)	1.00 (ref)
Other	2.55 (1.28–5.10)	0.46 (0.23–0.94)*	0.55 (0.27–1.14)
Deprivation status			
1 (least deprived)	5.78 (4.29–7.79)	1.00 (ref)	1.00 (ref)
2	5.42 (3.88–7.59)	0.94 (0.60–1.47)	0.95 (0.60–1.50)
3	5.18 (3.62–7.42)	0.90 (0.56–1.43)	0.82 (0.51–1.34)
4	4.07 (2.70–6.12)	0.70 (0.42–1.17)	0.76 (0.46–1.27)
5 (most deprived)	4.39 (2.89–6.67)	0.76 (0.45–1.27)	0.78 (0.46–1.33)
*No long-term condition group*			
Age	NA	1.12 (1.09–1.17)**	1.13 (1.09–1.18)**
Sex			
Male	2.20 (1.78–2.72)	1.00 (ref)	1.00 (ref)
Female	3.70 (3.07–4.47)	1.69 (1.27–2.24)**	1.77 (1.30–2.41)**
Ethnicity			
White	2.97 (2.53–3.48)	1.00 (ref)	1.00 (ref)
Other	2.63 (1.56–4.45)	0.89 (0.51–1.53)	1.03 (0.59–1.80)
Deprivation status			
1 (least deprived)	3.14 (2.45–4.02)	1.00 (ref)	1.00 (ref)
2	2.65 (1.93–3.64)	0.84 (0.56–1.26)	0.84 (0.55–1.29)
3	3.15 (2.30–4.31)	1.00 (0.67–1.50)	0.82 (0.53–1.29)
4	2.34 (1.60–3.41)	0.74 (0.47–1.17)	0.69 (0.43–1.12)
5 (most deprived)	2.81 (1.94–4.07)	0.90 (0.57–1.40)	0.81 (0.50–1.32)

Adjusted incidence rate ratio adjusted for age, sex, ethnicity, and deprivation status (as appropriate).

*NA* not applicable.

*p ≤ 0.05; **p ≤ 0.01.

### Incidence of depression

Of the 25,313 children and young people in the study sample, 51 had been diagnosed with depression prior to the start of the study. Therefore, 25,262 children and young people were included in the analysis of depression incidence, providing a total follow-up time of 122,750.48 person-years (median follow-up time: 4.59 (IQR: 2.21–7.24) years). In total, 393 (1.56%) children and young people had an incident case of depression (Table 3). Less than two-thirds of children and young people with incident depression (59.3%) had a diagnostic depression code recorded (Fig. 3b and Table 4).

**Table 4:** Incidence of depression, by condition group.

	Number of children	Depression cases, no. (%)	Person-years in 1000s	Depression incidence rate per 1000 person-years (95% CI)	Crude incidence rate ratio (95% CI)	Adjusted incidence rate ratio (95% CI)
Total sample	25,262	393 (1.56)	122.75	3.20 (2.90–3.53)	NA	NA
Condition group						
No long-term condition	13,049	153 (1.17)	68.63	2.23 (1.90–2.61)	1.00 (ref)	1.00 (ref)
Chronic condition	6707	148 (2.21)	30.34	4.88 (4.15–5.73)	2.19 (1.75–2.74)**	1.81 (1.44–2.28)**
Life-limiting condition	5506	92 (1.67)	23.78	3.87 (3.15–4.75)	1.74 (1.34–2.25)**	1.41 (1.08–1.83)*

Adjusted incidence rate ratio adjusted for age, sex, ethnicity, and deprivation status.

*NA* not applicable.

*p ≤ 0.05; **p ≤ 0.01.

The highest crude incidence of depression was observed in the chronic condition group (4.88 cases per 1000 person-years, 95% CI: 4.15–5.73), followed by the life-limiting condition group (3.87 cases per 1000 person-years, 95% CI: 3.15–4.75). The lowest incidence of depression was observed in the no long-term condition group (2.23 cases per 1000 person-years, 95% CI: 1.90–2.61) (Fig. 4b). Both the crude and adjusted depression incidence rate ratios were significantly higher in the life-limiting condition group (IRR_adj_: 1.41, 95% CI: 1.08–1.83) and the chronic condition group (IRR_adj_: 1.81, 95% CI: 1.44–2.28) compared to the no long-term condition group (Table 5). The full results from the adjusted model can be found in Supplementary Table 4.

**Table 5:** Stratified analysis of potential risk factors for depression incidence, by condition group.

	Incidence rate (per 1000 person-years) (95% CI)	Crude incidence rate ratio (95% CI)	Adjusted incidence rate ratio (95% CI)
*Life-limiting condition group*			
Age	NA	1.28 (1.21–1.34)**	1.27 (1.21–1.34)**
Sex			
Male	2.67 (1.93–3.68)	1.00 (ref)	1.00 (ref)
Female	5.55 (4.26–7.23)	2.08 (1.37–3.16)**	2.15 (1.41–3.27)**
Ethnicity			
White	4.28 (3.47–5.27)	1.00 (ref)	1.00 (ref)
Other	1.17 (0.38–3.62)	0.27 (0.09–0.86)*	0.36 (0.11–1.15)
Deprivation status			
1 (least deprived)	3.64 (2.35–5.63)	1.00 (ref)	1.00 (ref)
2	3.24 (2.01–5.21)	0.89 (0.47–1.70)	0.80 (0.42–1.53)
3	3.53 (2.16–5.76)	0.97 (0.50–1.87)	0.86 (0.44–1.65)
4	3.37 (2.03–5.59)	0.3 (0.47–1.81)	0.85 (0.43–1.66)
5 (most deprived)	5.95 (3.99–8.88)	1.64 (0.90–2.96)	1.53 (0.84–2.80)
*Chronic condition group*			
Age	NA	1.26 (1.21–1.31)**	1.25 (1.20–1.30)**
Sex			
Male	3.39 (2.63–4.37)	1.00 (ref)	1.00 (ref)
Female	6.96 (5.65–8.58)	2.05 (1.48–2.85)	2.02 (1.44–2.84)**
Ethnicity			
White	5.25 (4.43–6.24)	1.00 (ref)	1.00 (ref)
Other	2.54 (1.27–5.09)	0.48 (0.24–0.99)*	0.63 (0.31–1.30)
Deprivation status			
1 (least deprived)	5.60 (4.14–7.58)	1.00 (ref)	1.00 (ref)
2	3.97 (2.68–5.87)	0.71 (0.43–1.16)	0.71 (0.43–1.18)
3	4.46 (3.04–6.55)	0.80 (0.49–1.30)	0.79 (0.48–1.31)
4	4.22 (2.83–6.29)	0.75 (0.46–1.24)	0.80 (0.48–1.35)
5 (most deprived)	6.19 (4.35–8.80)	1.10 (0.69–1.76)	1.15 (0.71–1.87)
*No long-term condition group*			
Age	NA	1.26 (1.21–1.31)**	1.27 (1.22–1.33)**
Sex			
Male	1.26 (0.95–1.67)	1.00 (ref)	1.00 (ref)
Female	3.49 (2.88–4.23)	2.77 (1.97–3.89)**	2.87 (2.01–4.10)**
Ethnicity			
White	2.61 (2.20–3.09)	1.00 (ref)	1.00 (ref)
Other	0.56 (0.18–1.74)	0.22 (0.07–0.68)**	0.30 (0.10–0.96)*
Deprivation status			
1 (least deprived)	2.43 (1.84–3.21)	1.00 (ref)	1.00 (ref)
2	2.08 (1.46–2.98)	0.86 (0.54–1.35)	0.82 (0.51–1.32)
3	2.66 (1.89–3.74)	1.09 (0.70–1.70)	0.96 (0.60–1.52)
4	2.07 (1.39–3.09)	0.85 (0.52–1.39)	0.77 (0.46–1.28)
5 (most deprived)	1.70 (1.06–2.74)	0.70 (0.40–1.22)	0.55 (0.30–1.02)

Adjusted incidence rate ratio adjusted for age, sex, ethnicity, and deprivation status (as appropriate).

*NA* not applicable.

*p ≤ 0.05; **p ≤ 0.01.

In all three condition groups, the incidence of depression in females was over double that observed in males (IRR_adj_ comparing females to males for life-limiting condition group: 2.15, 95% CI: 1.41–3.27; IRR_adj_ for chronic condition group: 2.02, 95% CI: 1.44–2.84; IRR_adj_ for no long-term condition group: 2.87, 95% CI: 2.01–4.10). Increased age was also significantly associated with depression incidence across all three condition groups. Although not statistically significant, a higher incidence of depression was observed among children and young people from the Other ethnic group, compared to the White ethnic group, in all condition groups. No association was observed between deprivation status and depression incidence in any of the condition groups (Table 5).

## Discussion

This population-based study has shown that the incidence of both anxiety and depression were significantly higher among children and young people with life-limiting conditions and chronic conditions, compared to those with no long-term conditions. Despite the wide range of life-limiting conditions included in this study, the findings still align with results from previous literature, which indicated a higher prevalence of anxiety and depression among children and young people with specific life-limiting conditions, compared to those in the general population.^18^ The finding that chronic conditions appear to present a greater risk for anxiety and depression than life-limiting conditions may be due to a number of factors and certainly merits further investigation. One potential explanation for this finding is the possible under-recognition of psychological distress in children and young people with intellectual disabilities, which are far more prevalent among children and young people with life-limiting conditions compared to chronic conditions.^26,28^ In addition, HES data does not systematically report psychological interventions delivered within the context of multi-disciplinary tertiary care, potentially resulting in further under-reporting of anxiety and depression in children and young people with life-limiting conditions, who are more likely to be regularly present within the tertiary care system.

This study also showed that female sex and older age increased the risk of anxiety and depression in children and young people with life-limiting conditions, chronic conditions, or no long-term conditions, supporting findings from previous literature in the general population.^7,29,30^ In all three condition groups, a higher incidence of anxiety and depression was observed among children and young people from the Other ethnic group, compared to children and young people from White ethnic groups. This disparity in mental health conditions between White and minority ethnic groups has frequently been observed in previous literature, and may be the result of cultural differences in the perception and expression of symptoms relating to mental health conditions, which raises questions regarding the cultural appropriateness of diagnostic systems.^7,31,32^ Despite previous studies reporting significantly higher anxiety/depression incidence rates among individuals living in more deprived areas,^24,29,33^ no significant association between the incidence of anxiety/depression and deprivation status was observed in this study.

The increased risk of anxiety and depression observed among children and young people with life-limiting conditions highlights the importance of the promotion of psychological wellbeing and the prevention of mental health conditions. The identification of risk factors for anxiety and depression allows for preventative services to be targeted to those children and young people most at risk of developing these mental health conditions. It is also important that anxiety and depression can be rapidly identified. UK guidance for the care and support of individuals living with some life-limiting conditions (cystic fibrosis, cancer, cerebral palsy, and Duchenne muscular dystrophy) all recommend regular psychological screening.^34–38^ However, a pan-European evaluation of screening implementation among cystic fibrosis patients found that screening had only been implemented in 50% of paediatric cystic fibrosis centres in Europe.^39^ Here, time constraints were reported by staff to be one of the main barriers to the implementation of screening. In addition, the high prevalence of intellectual disability among children and young people with life-limiting conditions and the resulting limited communicative abilities means that standard diagnostic criteria are likely to be inappropriate for use with these individuals, meaning that mental health conditions could go undetected.^28,40–43^ Accordingly, a previous study investigating the risk of anxiety and depression among adults with cerebral palsy found a lower incidence of these mental health conditions among adults with intellectual disability compared to those without.^26^ It is important, therefore, that future research investigates the development and evaluation of methods to reliably assess mental health conditions among children and young people with intellectual disability. In turn, this will allow for a deeper understanding of the epidemiology of anxiety and depression across the whole population of children and young people with life-limiting conditions, including those with co-occurring intellectual disability.

Issues also exist surrounding the treatment of anxiety and depression in children and young people with life-limiting conditions. Although, in theory, either generic or specialist mental health services could be used to manage mental health conditions in this population, depending on the specific needs of the child or young person, the limited availability of both of these services in the UK presents a major barrier to the effective psychological care of this population.^39,44,45^

### Strengths and weaknesses

This was the first longitudinal study investigating the incidence of anxiety and depression in children and young people with life-limiting conditions, and therefore allowed the temporality of the association between the diagnosis of a life-limiting condition and the development of anxiety/depression to be analysed for the first time. The use of CPRD and HES data from a large, representative sample of children and young people also allowed for the inclusion of a wide range of life-limiting conditions, therefore increasing the generalisability of the study’s findings. Additionally, the analysis of primary and secondary care data from nationally representative and medically accurate data sources increased the validity and reliability of the study’s findings.^46–48^

The detailed nature of the data available within CPRD and HES, including various mental health indicators, allowed for the development of comprehensive algorithms to identify the presence of anxiety and depression, another major strength of this study. Previous research has shown that the most sensitive algorithms for the detection of anxiety and depression include indicators relating to diagnoses, symptoms, and treatment.^49^ Accordingly, the importance of the inclusion of all of these code types in the algorithms was highlighted by the findings from the current study, which showed that nearly 41% of depression cases would have been missed if identification relied on diagnostic codes alone.

However, the incidence of anxiety and depression calculated in this study only represents the children and young people who presented to primary or secondary care with anxiety or depression. Several factors can influence the likelihood of children and young people, or their parents, seeking medical help for potential mental health conditions, and therefore the anxiety and depression incidence rates presented in this study are likely to be underestimates of the incidence in the community.^50,51^ However, there is no evidence that this would have differed between the three condition groups. Therefore, this should not have affected  the validity of the comparison of incidence rates between children and young people with life-limiting conditions, chronic conditions or no long-term conditions. In addition, this study only looked at the presence or absence of anxiety and depression, rather than the severity of these mental health conditions. Therefore, future research should compare the severity of anxiety and depression across the three condition groups in order to further understand the impact of life-limiting/chronic conditions on the mental health of children and young people.

Additionally, some prescription data could not be utilised in this study; namely anxiolytics, hypnotics, and some antidepressants which are commonly prescribed for conditions other than anxiety or depression. Although this could have resulted in an underestimation of the incidence of anxiety and depression, the exclusion of this prescription data was necessary as several of the indications for which these drugs are used, such as neuropathic pain and seizures, are far more likely to occur in children and young people with life-limiting conditions. Therefore, the inclusion of these drugs may have generated biased incidence estimates which were not comparable across the three condition groups. Finally, the small group sizes used in the stratified analysis of ethnicity and deprivation status may have meant that the analysis was underpowered to detect existing associations.

## Conclusion

This study demonstrates that the incidence of anxiety and depression is significantly higher in children and young people with life-limiting conditions, compared to those with no long-term conditions. These findings highlight the need for increased attention to be paid to the mental health needs of this population, including strategies and interventions to prevent, identify, and treat anxiety and depression.

## Supplementary information

Supplementary Materials

## Data Availability

The patient-level data used in this study cannot be shared but can be accessed via CPRD.

## References

[1] Whiteford HA (2013). Global burden of disease attributable to mental and substance use disorders: findings from the Global Burden of Disease Study 2010. Lancet.

[2] Kieling C (2011). Child and adolescent mental health worldwide: evidence for action. Lancet.

[3] Kessler RC (2005). Lifetime prevalence and age-of-onset distributions of DSM-IV disorders in the National Comorbidity Survey Replication. Arch. Gen. Psychiatry.

[4] Polanczyk GV, Salum GA, Sugaya LS, Caye A, Rohde LA (2015). Annual research review: a meta‐analysis of the worldwide prevalence of mental disorders in children and adolescents. J. Child Psychol. Psychiatry.

[5] Pinquart M, Shen Y (2011). Anxiety in children and adolescents with chronic physical illnesses: a meta‐analysis. Acta. Paediatr..

[6] Pinquart M, Shen Y (2011). Depressive symptoms in children and adolescents with chronic physical illness: an updated meta-analysis. J. Pediatr. Psychol..

[7] Adams, J. S., Chien, A. T. & Wisk, L. E. Mental illness among youth with chronic physical conditions. *Pediatrics* **144**, e20181819 (2019). 10.1542/peds.2018-1819 PMC12930273 31201229

[8] Brady, A. M., Deighton, J. & Stansfeld, S. Chronic illness in childhood and early adolescence: a longitudinal exploration of co-occurring mental illness. *Dev. Psychopathol.* **33**, 885–898 (2020). 10.1017/S0954579420000206 32362290

[9] Together for Short Lives. A guide to the development of children’s palliative care. 4th edition. https://www.togetherforshortlives.org.uk/resource/a-guide-to-childrens-palliative-care/ (2018).

[10] Hedström M, Ljungman G, von Essen L (2005). Perceptions of distress among adolescents recently diagnosed with cancer. J. Pediatr. Hematol. Oncol..

[11] Greenley RN (2010). A meta-analytic review of the psychosocial adjustment of youth with inflammatory bowel disease. J. Pediatr. Psychol..

[12] Knight A, Vickery M, Fiks A, Barg F (2016). The illness experience of youth with lupus/mixed connective tissue disease: a mixed methods analysis of patient and parent perspectives. Lupus.

[13] Jamieson N (2014). Children’s experiences of cystic fibrosis: a systematic review of qualitative studies. Pediatrics.

[14] Fortier MA (2013). Illness uncertainty and quality of life in children with cancer. J. Pediatr. Hematol. Oncol..

[15] Namisango E (2019). Symptoms and concerns among children and young people with life-limiting and life-threatening conditions: a systematic review highlighting meaningful health outcomes. Patient.

[16] Higham L, Ahmed S, Ahmed M (2013). Hoping to live a “normal” life whilst living with unpredictable health and fear of death: impact of cystic fibrosis on young adults. J. Genet. Counseling.

[17] Fraser, L. K., Gibson-Smith, D., Jarvis, S., Norman, P. & Parslow, R. ‘Make every child count’ estimating current and future prevalence of children and young people with life-limiting conditions in the United Kingdom. https://www.york.ac.uk/healthsciences/research/public-health/projects/martinhouse/research/makeeverychildcount/ (2020).

[18] Barker MM, Beresford B, Bland M, Fraser LK (2019). Prevalence and incidence of anxiety and depression among children, adolescents, and young adults with life-limiting conditions: a systematic review and meta-analysis. JAMA Pediatr..

[19] Benchimol EI (2015). The reporting of studies conducted using observational routinely-collected health data (Record) statement. PLoS Med..

[20] CPRD. Data. https://cprd.com/Data (2020).

[21] Herrett E (2015). Data resource profile: Clinical Practice Research Datalink (CPRD). Int. J. Epidemiol..

[22] Fraser LK (2012). Rising national prevalence of life-limiting conditions in children in England. Pediatrics.

[23] Hardelid, P., Dattani, N. & Gilbert, R. Estimating the prevalence of chronic conditions in children who die in England, Scotland and Wales: a data linkage cohort study. *BMJ Open* **4**, e005331 (2014). 10.1136/bmjopen-2014-005331 PMC4127921 25085264

[24] Wijlaars LP, Nazareth I, Petersen I (2012). Trends in depression and antidepressant prescribing in children and adolescents: a cohort study in The Health Improvement Network (THIN). PLoS ONE.

[25] Davé S, Petersen I (2009). Creating medical and drug code lists to identify cases in primary care databases. Pharmacoepidemiol. Drug Saf..

[26] Smith KJ (2019). Risk of depression and anxiety in adults with cerebral palsy. JAMA Neurol..

[27] Department for Communities and Local Government. Official Statistics: English Indices of Deprivation 2010. https://www.gov.uk/government/statistics/english-indices-of-deprivation-2010 (2011).

[28] Feudtner C (2011). Pediatric palliative care patients: a prospective multicenter cohort study. Pediatrics.

[29] John A (2015). Recent trends in the incidence of anxiety and prescription of anxiolytics and hypnotics in children and young people: an E-Cohort Study. J. Affect. Disord..

[30] John A (2016). Case-finding for common mental disorders of anxiety and depression in primary care: an external validation of routinely collected data. BMC Med. Inform. Decis. Mak..

[31] Alegria M, Vallas M, Pumariega AJ (2010). Racial and ethnic disparities in pediatric mental health. Child Adolesc. Psychiatr. Clin..

[32] Liang J, Matheson BE, Douglas JM (2016). Mental health diagnostic considerations in racial/ethnic minority youth. J. Child Fam. Stud..

[33] John A (2016). Recent trends in primary-care antidepressant prescribing to children and young people: an E-Cohort Study. Psychol. Med..

[34] National Institute for Health and Care Excellence (NICE). Cystic fibrosis. https://www.nice.org.uk/guidance/qs55/chapter/Quality-statement-4-Psychological-and-social-support (2018).

[35] Quittner AL, Saez-Flores E, Barton JD (2016). The psychological burden of cystic fibrosis. Curr. Opin. Pulm. Med..

[36] National Institute for Health and Care Excellence (NICE). Cancer services for children and young people. https://www.nice.org.uk/guidance/qs55/chapter/Quality-statement-4-Psychological-and-social-support (2014).

[37] National Institute for Health and Care Excellence (NICE). Cerebral palsy in under 25s: assessment and management. https://www.nice.org.uk/guidance/ng62/chapter/recommendations (2017). 28151611

[38] Birnkrant DJ (2018). Diagnosis and management of duchenne muscular dystrophy, Part 3: Primary care, emergency management, psychosocial care, and transitions of care across the lifespan. Lancet Neurol..

[39] Abbott J (2019). Mental health screening in cystic fibrosis centres across Europe. J. Cyst. Fibros..

[40] Ekmekci, O. Pediatric multiple sclerosis and cognition: a review of clinical, neuropsychologic, and neuroradiologic features. *Behav. Neurol.* **2017**, 1463570 (2017). 10.1155/2017/1463570 PMC5757108 29434433

[41] Rae MG, O’Malley D (2016). Cognitive dysfunction in duchenne muscular dystrophy: a possible role for neuromodulatory immune molecules. J. Neurophysiol..

[42] Astrea G (2016). Learning disabilities in neuromuscular disorders: a springboard for adult life. Acta. Myologica.

[43] Maiano C (2018). Prevalence of anxiety and depressive disorders among youth with intellectual disabilities: a systematic review and meta-analysis. J. Affect. Disord..

[44] Garralda ME, Slaveska‐Hollis K (2016). What is special about a paediatric liaison child and adolescent mental health service?. Child Adolesc. Ment. Health.

[45] The Lancet. (2020). Child mental health services in England: a continuing crisis. Lancet.

[46] Herrett E, Thomas SL, Schoonen WM, Smeeth L, Hall AJ (2010). Validation and validity of diagnoses in the general practice research database: a systematic review. Br. J. Clin. Pharmacol..

[47] Burns EM (2012). Systematic review of discharge coding accuracy. J. Public Health.

[48] Campbell SE, Campbell MK, Grimshaw JM, Walker AE (2001). A systematic review of discharge coding accuracy. J. Public Health.

[49] Cornish, R. P., John, A., Boyd, A., Tilling, K. & Macleod, J. Defining adolescent common mental disorders using electronic primary care data: a comparison with outcomes measured using the CIS-R. *BMJ Open* **6**, e013167 (2016). 10.1136/bmjopen-2016-013167 PMC5168670 27909036

[50] Reardon T (2017). What do parents perceive are the barriers and facilitators to accessing psychological treatment for mental health problems in children and adolescents? A systematic review of qualitative and quantitative studies. Eur. Child Adolesc. Psychiatry.

[51] Radez, J. et al. Why do children and adolescents (not) seek and access professional help for their mental health problems? A systematic review of quantitative and qualitative studies. *Eur. Child Adolesc. Psychiatry* **30**, 183–211 (2020). 10.1007/s00787-019-01469-4 PMC7932953 31965309

