# Supplementary material for: Incidence of anxiety and depression in children and young people with life-limiting conditions
Source: Pediatr Res. 2022 Nov 11;93(7):2081–90. doi: 10.1038/s41390-022-02370-8 (PMC10313520; doi:10.1038/s41390-022-02370-8)
Supplement: Supplementary file 1 — Supplementary Materials [file 41390_2022_2370_MOESM1_ESM.docx]

**Supplementary Material**

**Supplementary Figure 1:** Diagram showing the process used to identify the study cohort

**Supplementary Table 1:** Decision of inclusion/exclusion for antidepressants

**Supplementary Table 2:** Decision of inclusion/exclusion for anxiolytics

**Supplementary Table 3:** Decision of inclusion/exclusion for hypnotics

**Supplementary Table 4:** Full results from multivariable model of anxiety incidence

**Supplementary Table 5:** Full results from multivariable model of depression incidence

***
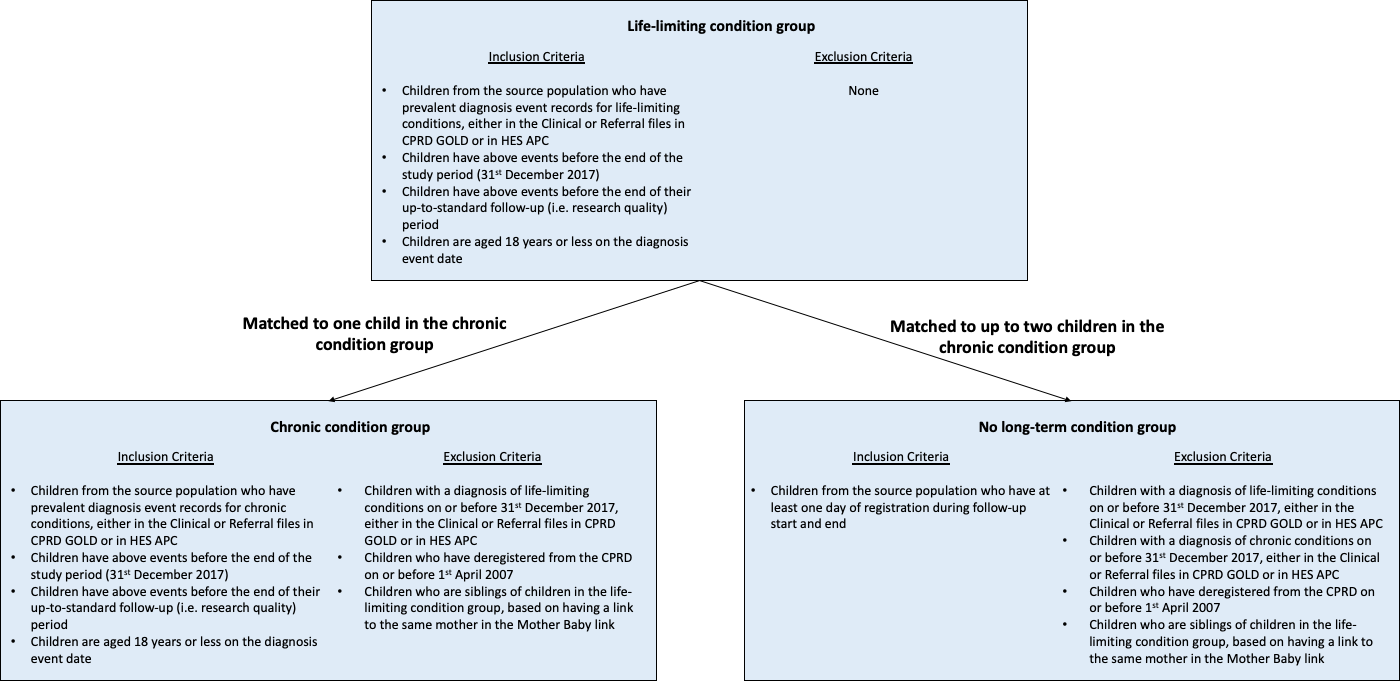
*Supplementary Figure 1:** Diagram showing the process used to identify the study cohort

Matched based on sex, year of birth, and region.

**Supplementary Table 1:** Decision of inclusion/exclusion for antidepressants

| **Antidepressant** | **Number of children and young people prescribed antidepressant** | | | **Decision** | **Other Indications** |
| --- | --- | --- | --- | --- | --- |
|  | **Life-limiting condition group** | **Chronic condition group** | **No long-term condition group** |  |  |
| **Amitriptyline** | 46 | 42 | 25 | Exclude | NA |
| record of depression | ≤10 | ≤10 | ≤10 |  |  |
| **Citalopram** | 22 | 46 | 48 | Include | None |
| record of depression | 16 | 34 | 29 |  |  |
| record of anxiety | 6 | 16 | 24 |  |  |
| **Clomipramine** | 0 | 0 | ≤10 | Include | None |
| record of depression | 0 | 0 | ≤10 |  |  |
| record of anxiety | 0 | 0 | ≤10 |  |  |
| **Dosulepin** | ≤10 | ≤10 | 0 | Include | None |
| record of depression | ≤10 | ≤10 | 0 |  |  |
| **Duloxetine** | ≤10 | ≤10 | ≤10 | Include | None |
| record of depression | ≤10 | ≤10 | 0 |  |  |
| record of anxiety | 0 | 0 | ≤10 |  |  |
| **Escitalopram** | ≤10 | ≤10 | ≤10 | Include | OCD |
| record of depression | 0 | ≤10 | ≤10 |  |  |
| record of anxiety | 0 | ≤10 | ≤10 |  |  |
| **Fluoxetine** | 37 | 44 | 45 | Include | OCD  Eating disorders |
| record of depression | 19 | 31 | 33 |  |  |
| record of anxiety | ≤10 | 14 | 18 |  |  |
| **Flupentixol** | 0 | 0 | ≤10 | Include | None |
| record of depression | 0 | 0 | 0 |  |  |
| **Fluvoxamine** | 0 | 0 | ≤10 | Include | OCD |
| record of depression | 0 | 0 | 0 |  |  |
| **Imipramine** | ≤10 | ≤10 | ≤10 | Exclude | NA |
| record of depression | 0 | 0 | 0 |  |  |
| **Lofepramine** | ≤10 | 0 | 0 | Include | None |
| record of depression | 1 | 0 | 0 |  |  |
| **Mirtazapine** | ≤10 | ≤10 | ≤10 | Include | None |
| record of depression | ≤10 | ≤10 | ≤10 |  |  |
| **Nortriptyline** | 0 | ≤10 | 0 | Include | None |
| record of depression | 0 | ≤10 | 0 |  |  |
| **Paroxetine** | ≤10 | 0 | ≤10 | Include | OCD |
| record of depression | ≤10 | 0 | 0 |  |  |
| record of anxiety | ≤10 | 0 | 0 |  |  |
| **Pregabalin** | ≤10 | ≤10 | 0 | Exclude | NA |
| record of depression | ≤10 | 0 | 0 |  |  |
| record of anxiety | 0 | 0 | 0 |  |  |
| **Sertraline** | 14 | 35 | 23 | Include | OCD |
| record of depression | ≤10 | 22 | 12 |  |  |
| record of anxiety | ≤10 | 17 | ≤10 |  |  |
| **Venlafaxine** | ≤10 | ≤10 | 0 | Include | None |
| record of depression | 0 | ≤10 | 0 |  |  |
| record of anxiety | 0 | 0 | 0 |  |  |

Shows the number of children who have a record of depression (and anxiety if antidepressant also indicated for anxiety) before the prescription, by condition group.

Displays the main other indications for included drugs

Cell values of 10 or less are censored (‘≤10’) and some cells with values greater than 10 are also censored to prevent censored cells being determined by differencing (^).

**Supplementary Table 2:** Decision of inclusion/exclusion for anxiolytics

| **Anxiolytic** | **Number of children and young people prescribed anxiolytic** | | | **Decision** |
| --- | --- | --- | --- | --- |
|  | **Life-limiting condition group** | **Chronic condition group** | **No long-term condition group** |  |
| **Buspirone** | ≤10 | 0 | ≤10 | Exclude |
| record of anxiety | 0 | 0 | 0 |  |
| **Clobazam** | 20 | ≤10 | 0 | Exclude |
| record of anxiety | ≤10 | 0 | 0 |  |
| **Diazepam** | 175 | 30 | 31 | Exclude |
| record of anxiety | 12 | 13 | ≤10 |  |
| **Hydroxyzine** | ≤10 | 0 | 0 | Exclude |
| record of anxiety | 0 | 0 | 0 |  |
| **Lorazepam** | 15 | ≤10 | 0 | Exclude |
| record of anxiety | ≤10 | 0 | 0 |  |
| **Midazolam** | ≤10 | 0 | 0 | Exclude |
| record of anxiety | ≤10 | 0 | 0 |  |

Shows the number of children and young people prescribed each anxiolytic and the number of these children who have a record of anxiety before the prescription, by condition group.

Cell values of 10 or less are censored (‘≤10’) and some cells with values greater than 10 are also censored to prevent censored cells being determined by differencing (^).

**Supplementary Table 3:** Decision of inclusion/exclusion for hypnotics

| **Hypnotic** | **Number of children and young people prescribed hypnotic** | | | **Decision** |
| --- | --- | --- | --- | --- |
|  | **Life-limiting condition group** | **Chronic condition group** | **No long-term condition group** |  |
| **Chloral hydrate** | 30 | ≤10 | ≤10 | Exclude |
| record of anxiety | ≤10 | 0 | ≤10 |  |
| **Chloral betaine** | ≤10 | 0 | 0 | Exclude |
| record of anxiety | 0 | 0 | 0 |  |
| **Hydroxyzine** | 14 | 16 | ≤10 | Exclude |
| record of anxiety | ≤10 | ≤10 | ≤10 |  |
| **Melatonin** | 216 | 104 | 26 | Exclude |
| record of anxiety | 17 | ≤10 | ≤10 |  |
| **Nitrazepam** | 13 | 0 | ≤10 | Exclude |
| record of anxiety | ≤10 | 0 | 0 |  |
| **Promethazine** | 61 | 73 | 48 | Exclude |
| record of anxiety | ≤10 | ≤10 | ≤10 |  |
| **Temazepam** | ≤10 | 0 | ≤10 | Exclude |
| record of anxiety | 0 | 0 | ≤10 |  |
| **Triclofos** | ≤10 | 0 | 0 | Exclude |
| record of anxiety | 0 | 0 | 0 |  |
| **Zopiclone** | ≤10 | 12 | ≤10 | Exclude |
| record of anxiety | ≤10 | ≤10 | ≤10 |  |

Shows the number of children and young people prescribed each hypnotic and the number of these children who have a record of anxiety before the prescription, by condition group.

Cell values of 10 or less are censored (‘≤10’) and some cells with values greater than 10 are also censored to prevent censored cells being determined by differencing (^).

**Supplementary Table 4:** Full results from multivariable model of anxiety incidence

|  | **Adjusted incidence rate ratio (95% CI)** |
| --- | --- |
| **Condition group** | |
| No long-term condition | 1.00 (ref) |
| Chronic condition | 1.64 (1.32-2.03)** |
| Life-limiting condition | 1.39 (1.09-1.77)** |
| **Age** | 1.13 (1.11-1.16)** |
| **Sex** |  |
| Male | 1.00 (ref) |
| Female | 1.86 (1.54-2.24)** |
| **Ethnicity** | |
| White | 1.00 (ref) |
| Black | 0.79 (0.35-1.79) |
| South Asian | 0.56 (0.26-1.18) |
| Other Asian | 0.75 (0.24-2.34) |
| Other | 0.39 (0.10-1.56) |
| Mixed | 1.27 (0.67-2.38) |
| Missing | 0.72 (0.52-0.10)* |
| **Deprivation Status** | |
| 1 (least deprived) | 1.00 (ref) |
| 2 | 0.83 (0.64-1.09) |
| 3 | 0.86 (0.65-1.13) |
| 4 | 0.70 (0.52-0.94)* |
| 5 (most deprived) | 0.84 (0.63-1.13) |

**p*≤0.05 ***p*≤0.01

**NA:** Not applicable

Adjusted incidence rate ratio adjusted for age, sex, ethnicity, and deprivation status

**Supplementary Table 5:** Full results from multivariable model of depression incidence

|  | **Adjusted incidence rate ratio (95% CI)** |
| --- | --- |
| **Condition group** | |
| No long-term condition | 1.00 (ref) |
| Chronic condition | 1.81 (1.44-2.28)** |
| Life-limiting condition | 1.41 (1.44-2.28)* |
| **Age** | 1.26 (1.23-1.29)** |
| **Sex** |  |
| Male | 1.00 (ref) |
| Female | 2.35 (1.92-2.89)** |
| **Ethnicity** | |
| White | 1.00 (ref) |
| Black | 0.47 (0.15-1.48) |
| South Asian | 0.28 (0.09-0.88)* |
| Other Asian | 1.26 (0.47-3.39) |
| Other | 0.00 (0.00-0.00) |
| Mixed | 0.65 (0.24-1.73) |
| Missing | 0.45 (0.30-0.68)** |
| **Deprivation Status** | |
| 1 (least deprived) | 1.00 (ref) |
| 2 | 0.75 (0.56-1.01) |
| 3 | 0.86 (0.64-1.16) |
| 4 | 0.79 (0.58-1.08) |
| 5 (most deprived) | 1.07 (0.79-1.44) |

**p*≤0.05 ***p*≤0.01

**NA:** Not applicable

Adjusted incidence rate ratio adjusted for age, sex, ethnicity, and deprivation status
